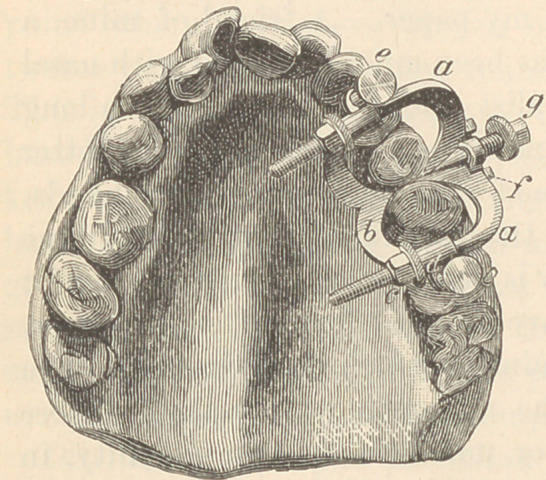# Central Dental Association of Northern New Jersey, Meeting at Newark, April 13, 1886

**Published:** 1886-06

**Authors:** 


### CENTRAL DENTAL ASSOCIATION OF NORTHERN NEW JERSEY, MEETING AT NEWARK, APRIL 13, 1886.

REPORTED EXPRESSLY FOR THE INDEPENDENT PRACTITIONER.

Dr. J. L. Osmun read a paper entitled "Suggestions in the Use of Nitrous Oxide Gas."

*Dr. W. H. Atkinson*—I think we could have no better argument to sustain my prejudice against the use of anæsthetics than the reading of this paper. It has not said one word about the necessity of administering nitrous oxide, or any other agent that will render any one insensible. That is the first step that should be taken into consideration. Is it necessary? I have yet to see the case where extraction of teeth was the object, when it was necessary. The use of it arises from the blackness of the darkness of ignorance that pertains to the anatomy and physiology and pathology of the teeth, and it is because of their familiarity with this abomination that men have not held it in the contempt which it deserves. The sin of the dental profession to-day is the extraction of teeth, and the insertion of the wretched substitutes which they laud to the skies. If you could have heard the experience of one of my patients, an old lady, as told in her own language, I am sure you would get down on your knees, with your hands in your mouths, and your mouths in the dust, and cry "unclean," before you would attempt to extract teeth as has been done in her case, and as is being done to-day.

*Dr. S. C. G. Watkins*—The giving of whisky, brandy, or any of the liquors, before administering gas, I believe increases the excitement and danger. Physicians, as a rule, when patients ask their advice in regard to taking gas, will advise them to take a glass of whisky first. In such cases, where I know they have taken liquor, I always send my patient away and tell him to come back in two or three hours, and then I give the gas, but never immediately after they have taken liquor, if I know it.

*Dr. C. S. Stockton*—I wish to reiterate what Dr. Watkins has said in regard to stimulants. I have seen the unfavorable effects of liquor taken before the administration of gas. I also believe with Dr. Atkinson, that it is rarely indeed that we should give an anæsthetic at all. With a little persuasion and perseverance a great many of our patients will yield, and when they do they are grateful to you afterwards that you did not give them an anæsthetic. In nine cases out of ten, where you have been compelled to inflict more or less pain, they will tell you they are glad they did not take the anæsthetic.

Sometimes we think the extraction of a pulp is about as painful a thing as we do in dentistry, and perhaps it is. In a number of instances, lately, I have been surprised to find how little pain it is necessary to inflict in order to remove a pulp. Our good friend, Dr. Pierce, once told me of a very troublesome patient of his who had a tooth in which the pulp had become exposed. He said he whittled a stick to a point and drove it into the canal with a mallet. To his great surprise the boy remained in the chair. He asked him if it did not hurt, and the boy replied that he felt it, and that was about all. I suppose that in two months' time I have removed the pulps of a dozen teeth in that way, and the patient hardly knew it had been done. I used no anæsthetic and no obtunder whatever.

*Dr. Atkinson*—It is very unfortunate for us that we follow fashion so much, and do not always select good fashions to follow. The description given of the knocking out of pulps is the very first method of destroying pulps that I was made acquainted with when I was a boy, when we used to set pivot teeth with wooden pivots. We whittled a stick to a point and drove it up with a mallet, then twirled it around. Many of the pulps so treated healed at the point where they were broken off. I have taken the pivots out twelve years afterwards, and found a remnant of the pulp living. This is only a new "discovery" of an old practice. If we could have had, at the time when I was a boy, societies for spreading the knowledge of what we were doing, rather than hiding it, it would have been better, and if we had more conscience and less desire to get unclean money we would not be running against each other to see who could get the "job" from the other at a less rate, and would not be extracting teeth to put in four dollar sets. It is the unclean money business that tempts men to their fall.

*Dr. Osmun*—In the discussion that has taken place, the impression seems to be that I am in favor of administering anæsthetics, and the extraction of teeth by wholesale. If I have given that impression, nothing was farther from my mind. I simply took up the subject as one full of interest. As gas is administered constantly, I thought it would be a good subject to talk about. I believe in the conservative treatment of teeth under all circumstances.

Dr. Stockton introduced Dr. Pritchard, of London.

Dr. Pritchard remarked, that when the millennium arrived, Dr. Atkinson's anti-anæsthetic ideas might prevail, and then he would be happy to meet him, but until then he thought it would be occasionally necessary to use an anæsthetic.

Dr. C. S. W. Baldwin read a paper on "Nasal Catarrh."

*Dr. Osmun*—I think that all treatment of nasal catarrh that employs warm applications is dangerous. A new instrument has been employed for treating nasal diseases. It is a compressed air cylinder, carrying from forty to fifty pounds' pressure to the square inch, and you apply the remedies cold, in the form of a fine spray. The compressed air gives sufficient force to carry the medicaments where it would be impossible to get them with steam. With the compressed air instrument a spray can be thrown at different angles and made to reach almost any part of the nasal tract. I think, however, it would be better for us to stick to the legitimate domain of dentistry. The specialist who covers our field completely has about all that he can attend to, and if he notices allied diseases it is better to send the cases to a specialist in that line, who has special appliances and special skill.

*Dr. Baldwin*—I am happy to hear a hearty expression of opinion, although it is in condemnation of my paper. A friend of mine, a doctor of divinity in New York, has been much troubled with nasal catarrh. He was sent to a specialist and was treated for a long time, and paid a large sum of money, and came away no better than he was before. It so happened that he came into my hands, and he was so much benefited by the treatment that he urged me to give it publicity. While theory is good, practice is better, and it has led me to the conclusions I have brought forward in the paper.

*Dr. Atkinson*—There is one point in which there seems to be a serious lack of apprehension of the underlying principles. There is what is called the hygrometric, or moisture-absorbing quality, in the mucous membrane of the nose. Those little epithelial bodies that lie upon the surface of the mucous membrane are a very crotchety set of fellows. Sometimes they will be so swelled up by the imbibition of water taken into the nostrils as to completely close the nares. You have often noticed, when lying on one side, that one of the nostrils would be closed and you could breathe only through the other, and that when you turned over the one that was open would close and the other one open. This is because gravity acts upon the surcharged epithelial bodies constituting the mucous membrane. Let us know the principles that underlie these circumstances. There is some sense in applying heat in such cases. When you want to wash anything you do not use very cold water, and you put something in it. For cleansing the nostrils use soap suds; not the mottled castile soap, but the white. Wash the nostril until it is free from dry mucus, then use some kind of an agent that will destroy the microbes, and after that vaseline. Never put pure water into the nostrils, nor into any other air passage. Modify it by adding a little salt, or chloride of sodium, until it is about as saline as sea water.

*Dr. Pinney*—Mr. President, I wish to introduce Dr. Parr, of New York, who has a little appliance that he would like to show you. He has applied it to my teeth, and I can say that it is the most complete little machine that I ever saw. Without any pain or trouble, it separated my teeth so far apart that they could be easily filled; and it was done in five minutes, and a greater space obtained than would have been obtained in three or four days with wedges.

*Dr. H. A. Parr*—The separator possesses the following advantages or qualities: First, it is universal in its application. It can be adjusted to the upper or the lower teeth, to molars, centrals or bicuspids equally well. Second, it is particularly adapted to irregular teeth. Third, it may be advantageously employed in the correction of many cases of irregularity. I will describe to you its different parts in detail, after which you can examine the instrument and the models. (See cut.) In this diagram *a* represents an angular bar which is tapered to a point, reaching out in a semicircular form, and has parallel sockets, *b*. *c c* are two semicircular bars, the inner ends of which are tapered to a point and meet at an acute angle, directly opposite the angle of the bar *a*. The arms of *c* are made long, and pass through the socket *b*, and have threads and nuts, *c* and *d*, which can be moved with the thumb and finger, or a wrench. Upon the convex sides of the bar *c* are lugs, *e*, to receive the movable cross-bar *f*, which is pierced by a screw, *g*, which screw has a conical end that rests between the bars *c*, and upon being turned forces the bars *c* apart, thereby effecting the desired separation. Under ordinary circumstances the turning of the nuts *d*, which may be used on either buccal or lingual surfaces, as may be desired, will be sufficient; but when more power is required, *g* should be used. *h h* are wings through which pass screws, *i*, by which the instrument may be adjusted to teeth of any length and prevent undue pressure on the gums.

*Dr. Palmer*—The one particular feature of Dr. Parr's separator that I like is its adaptability to any case of irregularity. In cases where the eye teeth are very prominent, or the lateral impinges on the central and canine, this separator will perform the work as well as if the teeth stood in proper position. In regulating teeth this instrument will move them in a few hours, where you might take days by other methods, and not do it as successfully.

## Figures and Tables

**Figure f1:**